# Diving in the Arctic: Cold Water Immersion’s Effects on Heart Rate Variability in Navy Divers

**DOI:** 10.3389/fphys.2019.01600

**Published:** 2020-01-31

**Authors:** Richard V. Lundell, Anne K. Räisänen-Sokolowski, Tomi K. Wuorimaa, Tommi Ojanen, Kai I. Parkkola

**Affiliations:** ^1^Diving Medical Centre, Centre for Military Medicine, The Finnish Defence Forces, Helsinki, Finland; ^2^Doctoral Programme in Clinical Research, University of Helsinki, Helsinki, Finland; ^3^Department of Pathology, HUSLAB, Helsinki University Hospital, University of Helsinki, Helsinki, Finland; ^4^Centre for Military Medicine, The Finnish Defence Forces, Kirkkonummi, Finland; ^5^Diving Medical Centre, Centre for Military Medicine, The Finnish Defence Forces, Kirkkonummi, Finland; ^6^Human Performance Division, Finnish Defence Research Agency, The Finnish Defence Forces, Tuusula, Finland; ^7^Faculty of Medicine and Health Technology, Tampere University, Tampere, Finland; ^8^National Defence University, Helsinki, Finland

**Keywords:** diving reflex, diving response, sympathetic response, parasympathetic response, Arctic diving, cold water immersion

## Abstract

**Introduction:**

Diving close to the Arctic circle means diving in cold water regardless of the time of year. The human body reacts to cold through autonomous nervous system (ANS)-mediated thermoregulatory mechanisms. Diving also induces ANS responses as a result of the diving reflex.

**Materials and Methods:**

In order to study ANS responses during diving in Arctic water temperatures, we retrospectively analyzed repeated 5-min heart rate variability (HRV) measures and the mean body temperature from dives at regular intervals using naval diving equipment measurement tests in 0°C water. Three divers performed seven dives without physical activity (81–91 min), and two divers performed four dives with physical activity after 10 min of diving (0–10 min HRV recordings were included in the study).

**Results:**

Our study showed a significant increase in parasympathetic activity (PNS) at the beginning of the dives, after which PNS activity decreased significantly (measure 5–10 min). Subsequent measurements (15–20 min and onward) showed a significant increase in PNS activity over time.

**Conclusion:**

Our results suggest that the first PNS responses of the human diving reflex decrease quickly. Adverse effects of PNS activity should be considered on long and cold dives. To avoid concurrent sympathetic (SNS) and PNS activity at the beginning of dives, which in turn may increase the risk of arrhythmia in cold water, we suggest a short adaptation phase before physical activity. Moreover, we suggest it is prudent to give special attention to cardiovascular risk factors during pre-dive examinations for cold water divers.

## Introduction

Arctic water conditions induce special risk factors for divers. At a depth of 20 meters or deeper, water temperatures are 4°C throughout the year in Finland. For 4 months per year, the surface of the water is frozen, and, just below the layer of ice, water temperatures vary from −2 to 0°C in saltwater. Heat loss is uncomfortable for the divers, but it also impairs physical and cognitive performance (Davis et al., 1975; Bridgman, 1990), increases the risk of decompression illness (DCI), (Gerth, 2015; Pendergast et al., 2015) and may lead to hypothermia. These factors increase the risk of fatal diving accidents.

Both recreational and occupational divers in Northern Europe dive throughout the year. Even with the best efforts to keep divers warm, heat loss is unavoidable during Arctic diving. The human body reacts to cold through autonomous nervous system (ANS)-mediated thermoregulation mechanisms, (Morrison, 2016) such as vasoconstriction and shivering. The ANS responses to cold are well understood, but, to the best of our knowledge, there is no published data on these responses when measured during diving in Arctic water temperatures.

The aim of this study was to evaluate ANS responses in Finnish Navy divers when diving in 0°C water and with special emphasis on the first responses of the diving reflex. To achieve this, we analyzed short-term recordings (5 min) of heart rate variability (HRV) as values for ANS activity as well as mean body temperature (MBT) as a measure of body thermal balance during diving.

## Materials and Methods

### Subjects

The demographics of the Navy divers are shown in Table 1 and Supplementary Table S1. All divers (*n* = 4) volunteered for the study and gave their written consent. The data was collected during regular Naval diving equipment development tests and analyzed retrospectively. Approval from the Ethical Committee of Tampere University Hospital was obtained. The study protocol was accepted by The Logistic Department of the Defence Command Finland (2018), and it adhered to the Declaration of Helsinki.

**TABLE 1:** Demographics of the four study subjects.

**Age in years**	**Height in meters**	**Weight in kg**	**BMI in kg/m^2^**	**Body Fat Mass in kg**	**Body Muscle Mass in kg**
**range and (mean)**	**range and (mean)**	**range and (mean)**	**range and (mean)**	**range and (mean)**	**range and (mean)**
25 – 43	1.78 – 1.81	79.2 – 86.8	24.8 – 29.2	4.9 – 14.5	37.2 – 43.3
(39)	(1.78)	(83.2)	(26.4)	(11.5)	(41.2)

### Diving Equipment

The divers used standard military SCUBA diving equipment for Arctic conditions: standard regulator mask, standard dry suit, standard diving hood, standard diving gloves, standard diving underwear, 70% merino wool socks, and elbow and knee warmers. In addition to this, the divers wore Merinot 100% merino wool polo skirts and pants. All divers used air as their breathing gas.

### Diving Procedure

The tests were performed over the course of 3 days in January near the Arctic Circle. Four divers performed 11 dives in total during the tests [dives for each diver (D1–D4)]: D1 two dives, D2 three dives, and D3 three dives (of these, one 0–10 min dive was included in study because after this the diver could move freely and physical activity was not controlled), D4 three dives (all three 0–10 min dives were included in study because after this the diver could move freely and physical activity was not controlled). Subjects dived only once a day. During the diving days the divers had their normal 6–8 h a night of sleep, and no exercise was permitted for the 4 h prior to the dives.

During the tests, air temperature varied from −13 to −24°C. Water temperature in the diving depth was 0°C during all dives. Diving equipment and sensors were donned with the assistance of staff members in a consistent room temperature of 18–19°C. After this, the divers walked about 30 meters to the river where an ice hole had been bored and performed their dive without further delay. The participants dived to the bottom of the river to a depth of 6 meters (160 kPa) where they remained still in a horizontal prone position for 80–91 min (*n* = 7). Other dives (*n* = 4) had the same protocol at the beginning of the dives (0–10 min). After this, staying still was not required, and the amount of physical activity was therefore not controlled.

### Measurements

Divers’ fat and muscle mass were measured with the InBody 720 composition analyzer (Biospace Ltd., Seoul, South Korea).

We recorded the heart rate (R to R wave measures at a 1000 Hz sampling frequency) with the HRV Bodyguard device (Firstbeat Technologies Ltd., Jyväskylä, Finland). Using these recordings, the HRV was analyzed with the Kubios HRV Standard program (Ver. 3.1, Kubios Ltd., Kuopio, Finland) from the recordings on diving subjects for the following measurement time intervals (M1–M9 and MR). We used the program’s automatic artifact correction to correct corruption in data and the program’s time series trend removal tool for each subject before analysis (Lipponen and Tarvainen, 2019). The start of the dive is defined as time point 0 min:

M1 0–5 min (11 dives),

M2 5–10 min (11 dives),

M3 15–20 min (7 dives),

M4 25–30 min (7 dives),

M5 35–40 min (7 dives),

M6 45–50 min (7 dives),

M7 55–60 min (7 dives),

M8 65–70 min (7 dives),

M9 75–80 min (7 dives).

MR: 5-min recordings for each diving day of HRV in the morning at 0600 and in the evening at 2400 to get a HRV baseline at rest (MR) (2 × 11 measures = 22).

We used three time-domain measures and five frequency-domain measures.

Time-domain measures were recorded: (a) Mean heart rate (HR_mean_) (bpm), (b) Standard deviation of NN intervals (SDNN) (ms), and (c) Root mean square of successive RR interval differences (RMSSD) (ms).

Frequency-domain measures were recorded: (d) Absolute total power (TP) (ms^2^), (e) Absolute power of the very low frequency band (VLF) (ms^2^), (f) Absolute power of the low frequency band (LF) (ms^2^), (g) Absolute power of the high frequency band (HF) (ms^2^), and (h) Ratio of LF to HF power (LF/HF) (%).

For all 81–91 min of diving (*n* = 7), we also recorded deep body temperature (Trect), measured rectally with the Data Storage Tags (DST) sensor (Star-Oddi Ltd., Gardabaer, Iceland), and skin temperature (T_skin_), measured with the Smartreader Plus 8-system (ACR Systems Inc., Vancouver, BC, Canada) from eight standardized skin spots (left calf, right anterior thigh, right scapula, left upper chest, forehead, right arm in upper location, left arm in lower location, and left hand) (The International Organization for Standardization, 2004), for the whole duration of the dives.

We used the temperature values at the beginning of the HRV measurements (M1 0 min, M2 5 min, M3 15 min, M4 25 min, M5 35 min, M6 45 min, M7 55 min, M8 65 min, and M9 75 min) and calculated for each time point the MBT with Burtons formula:

$$M\,B\,T=\text{Trect}\times 0,65+\text{Tskin}\times 0,35^{9}$$

where T_rect_ is deep body temperature and T_skin_ is area weighted skin temperature (Burton, 1935).

T_skin_ was calculated with the ISO-standard weighting coefficients [0,2 × left calf, 0,19 × right anterior thigh, 0,175 × (right scapula + left upper chest), 0,07 × (forehead + right arm in upper location + left arm in lower location), 0,05 × left hand] (The International Organization for Standardization, 2004).

Interpretation of used HRV-measures:

(a) Heart rate was regulated by ANS input to the sinoatrial node. Sympathetic activity increased the heart while parasympathetic activity decreased the heart rate (Schmidt-Nielsen, 1997).
(b) Both the sympathetic nervous system and parasympathetic nervous system activity contributes to the SDNN. In short-term recordings, as in this project, the greatest source of the SDNN was the parasympathetically mediated respiratory sinus arrhythmia (Shaffer et al., 2014).
(c) The RMSSD illustrated the variance in the beat-to-beat heart rate and was the golden standard HRV measure for vagally mediated changes (Shaffer et al., 2014).
(d) The total power is the sum of power of ultra-low frequency (ULF), VLF, LF, and HF bands (Shaffer et al., 2014). An increase in T power was linked with parasympathetic activity, whereas a decrease was mostly seen as a result of sympathetic activity.
(e) The VLF band (0.0033–0.04 Hz) was influenced by many factors. The intrinsic nervous system of the heart seemed to contribute to it (Shaffer et al., 2014). Moreover, physical activity, thermoregulatory, renin-angiotensin, and endothelial influences on the heart contributed to it (Akselrod et al., 1981; Claydon and Krassioukov, 2008). PNS activity contributed strongly to VLF power (Taylor et al., 1998).
  The LF band (0.04–0.15 Hz) was produced by both the SNS and the PNS (Akselrod et al., 1981; Heart Rate Variability, 1996; Berntson et al., 2007). It also reflected baroreceptor activity in resting individuals (McCraty and Shaffer, 2015), primarily PNS activity via baroreceptors (Reyes del Paso et al., 2013) or baroreflex activity alone (Moak et al., 2007) contributed to LF power. Slow respiration rates, especially when one takes a deep breath or sighs, may have, through vagal activity, contributed to the LF band (Ahmed et al., 1982; Brown et al., 1993; Tiller et al., 1996; Lehrer et al., 2003).
(f) The HF band (0.15–0.40 Hz), also called the respiratory band, reflected parasympathetic activity and corresponded to heart rate variations related to the respiratory sinus arrhythmia (Grossman and Taylor, 2007). Low HF power was correlated with stress, worry, or anxiety.
(g) Under controlled conditions, LF/HF has been used to estimate the relation between the SNS and the PNS activity. In fact, as great portions of the LF band power is caused by the PNS and baroreceptor activity and smaller portions by other factors, the use of this ratio is challenged (Pagani et al., 1984, 1986). Also, SNS contribution to the LF band varies greatly depending on different testing conditions (Eckberg, 1983; Kember et al., 2001; Shaffer et al., 2014).

### Statistics

For evaluating changes, a linear mixed-effects model was performed. The analyses were performed with the R program (R Core Team, 2014).

Heart rate variability analyses were performed for all subjects for the beginnings of dives MR–M1 and M1–M2. For the 81–91 min of diving (*n* = 7), analyses were performed for M2–M9. Temperature analyses (*n* = 7) were performed for M1–M9, except for T_rect_, which seemed to show an increase from M1–M3 (M1–M3 and M3–M9 were analyzed separately).

## Results

**FIGURE 1:**
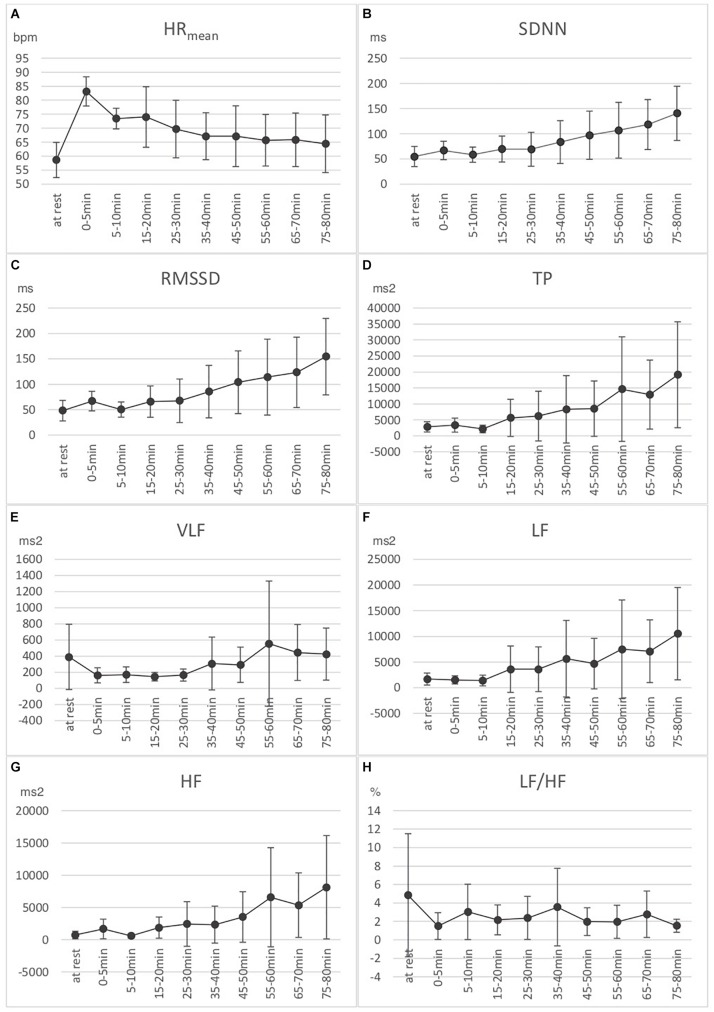
Eight HRV measures: three time-domain measures [**(A)** Mean heart rate (HR_mean_), **(B)** Standard deviation of NN intervals (SDNN), and **(C)** Root mean square of successive RR interval differences (RMSSD)], and five frequency-domain measures [**(D)** Absolute power of the very low frequency band (VLF power), **(E)** Absolute power of the low frequency band (LF), **(F)** Absolute power of the high frequency band (HF), **(G)** Absolute total power (TP), and **(H)** Ratio of LF to HF power (LF/HF)]. Measures are presented at rest (*n* = 22), 0–5 min (*n* = 11), 5–10 min (*n* = 11), 15–20 min (*n* = 7), 25–30 min (*n* = 7), 35–40 min (*n* = 7), 45–50 min (*n* = 7), 55–60 min (*n* = 7), 65–70 min (*n* = 7), and 75–80 min (*n* = 7). Means and standard errors shown.

**FIGURE 2:**
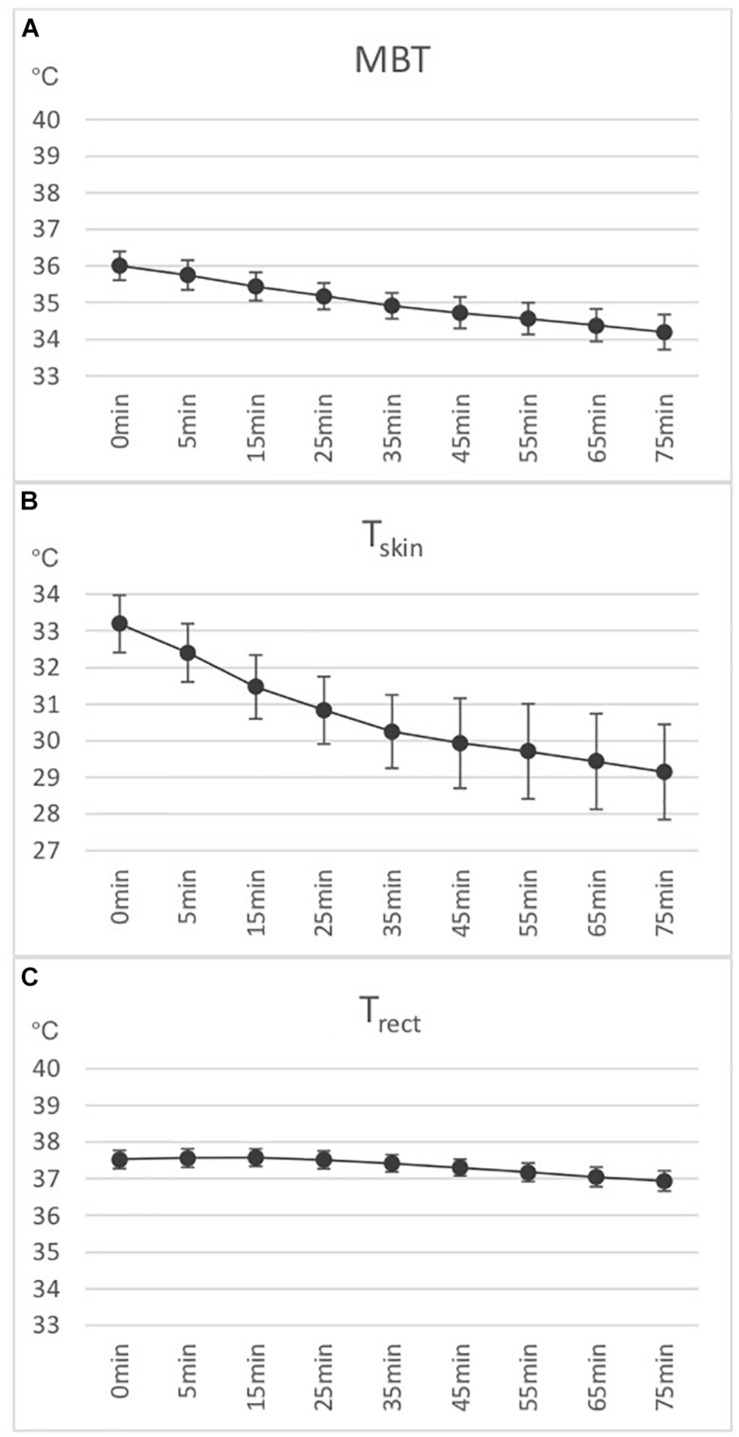
**(A)** Calculated mean body temperature (MBT) (*n* = 7). **(B)** Area weighted skin temperature (T_skin_) (*n* = 7). **(C)** Rectally measured deep body temperature (T_rect_) (*n* = 7). Means and standard errors shown.

Results for various HRV parameters are shown in Figures 1A–H. Temperature measures are shown in Figures 2A–C.

HRV measures:

(a) HR_mean_: From MR–M1, the heart rate increased significantly of 25 bpm (Standard error (SE) = 1.86, *p* < 0.001). From M1–M2, the heart rate decreased significantly of 9.08 bpm (SE = 1.86, *p* < 0.001). From M2–M9 (*n* = 7), the heart rate decreased significantly of 9.66 bpm (SE = 0.36, *p* < 0.001).
(b) SDNN: From MR–M1, there was a significant increase of 12.22 ms (SE = 5.79, *p* = 0.04). From M1–M2, there was a non-significant decrease of 6.99 ms (SE = 5.79, *p* = 0.23). From M2–M9 (*n* = 7), there was a significant increase of 78.61 ms (SE = 1.87, *p* < 0.001).
(c) RMSSD: From MR–M1, there was a significant increase of 14.67 ms (SE = 6.09, *p* = 0.02). From M1–M2, there was a significant decrease of 12.92 ms (SE = 6.09, *p* = 0.04). From M2–M9 (*n* = 7), there was a significant increase of 97.86 ms (SE = 2.28, *p* < 0.001).
(d) TP: From MR-M1 and M1–M2, there was no significant change. From M2–M9 (*n* = 7), there was a significant increase of 15044.61 ms^2^ (SE = 441.67, *p* < 0.001).
(e) VLF: From MR–M1, there was a significant decrease of 172.90 ms^2^ (SE = 60.98, *p* = 0.008). From M1–M2, there was no significant change (increase 6.27 ms^2^, SE = 60.98, *p* = 0.91). From M2–M9, there was no linear significant change.
(f) LF: From MR-M1 and M1–M2, there was no significant change. From M2–M9 (*n* = 7), there was a significant increase of 7675.92 ms^2^ (SE = 273.75, *p* < 0.001).
(g) HF: From MR–M1, there was a near-significant increase of 678.63 ms^2^ (SE = 344.48, *p* = 0.059). From M1–M2, there was a significant decrease of 835.73 ms^2^ (SE = 344.48, *p* = 0.022). From M2–M9, there was a significant increase of 6983.41 ms^2^ (SE = 208.23, *p* < 0.001).
(h) LF/HF: From MR–M1, there was a near-significant decrease of 2.46% (SE = 1.45, *p* = 0.10). From M1–M2, there was a significant increase of 3.05% (SE = 1.45, *p* = 0.045). From M2–M9 (*n* = 7), there was no significant linear change.

Temperatures:

(a) T_skin_: From M1–M9, there was a significant decrease of 3.92°C (SE = 0.05, *p* < 0.001).
(b) T_rect_: From M1–M3, there was a non-significant increase of 0.051°C (SE = 0.068, *p* = 0.46). From M3–M9, there was a significant decrease of 0.66°C (SE = 0.009, *p* < 0.001).
(c) MBT: From M1–M9, there was a significant decrease of 1.84°C (SE = 0.02, *p* < 0.001).

## Discussion

The novelty of our study is the observation that in these freezing water conditions, after a quick increase at the beginning of the dives, parasympathetic (PNS) activity actually decreased for HRV measures at 5–0 min. The first PNS response (M1) could be explained with a strong diving reflex at the beginning of the dives (Konishi et al., 2016; Schaller et al., 2017; Vega, 2017; McCulloch et al., 2018; Schlader et al., 2018). The next measure (M2) may suggest that, in humans, the diving reflex-induced PNS response actually decreased after a while. To the best of our knowledge, this finding has not been described in earlier studies. Our hypothesis is that the trigeminocardiac part of the diving reflex was lost quickly while the baroreceptor- and body temperature-induced PNS responses increased more slowly. This, most likely, has not been noticed because of other ANS responses covering the decrease in the trigeminocardiac part of the diving reflex, longer measurement intervals, and the usage of heart rate as the only measure. As we used short heart rate variability measures (5-min HRV) with a close measurement interval, the decrease in parasympathetic activity could be observed. Otherwise, for example, the increase in parasympathetic activity during immersion, correlates well with earlier observations from HRV studies in diving (Lund et al., 2000; Schipke and Pelzer, 2001; Kurita et al., 2002; Chouchou et al., 2009; Flouris and Scott, 2009; Noh et al., 2018).

After 15 min, PNS activity shown in HRV measures most likely increased because of hemodynamic changes through baroreceptors and a decrease in body temperature. The deep body temperature increased or stayed the same (no significant change) from 0 to 15 min because of a centralization of the blood volume. Both the hyperbaric pressure and the body’s thermoregulatory mechanisms contributed to this. MBT is a good measure for the heat loss of the body. It is a combination of both surface and deep body temperature, and it takes into account changes in blood redistribution. Cold is a known promoter of PNS activity (Vesoulis et al., 2017; Hodges et al., 2019). Since MBT decreased during the dives we would assume that temperature is an important factor in inducing the increase in PNS activity over time. Hyperbaric pressure was constant during dives since the divers were at a depth of six meters for the entire duration of the dives. Based on earlier studies, one could speculate that a change in PNS activity would be more dependent on the pressure and not as much on time in hyperbaric conditions (Barbosa et al., 2010). Only limited knowledge on the topic is available. On the other hand, throughout the dives we have seen a strong increase in the power of the LF-band, which would suggest ongoing baroreceptor activity. This would support the hypothesis of pressure-induced PNS activity over time. After the previously described short decrease in PNS activity at the beginning of the dives, PNS activity increased, according to our measurements, up to 80 min of diving (our last measurement). Our study did not determine how long diving in similar conditions induced PNS activity for. In theory, strong PNS activity may have possible adverse effects that could jeopardize diving safety, for example through an atrioventricular block, arrhythmia, syncope, or even sudden death (Aste and Brignole, 2017; Vaseghi et al., 2017; Japundzic-Zigon et al., 2018; Benito-Gomez et al., 2019).

When estimating the SNS activity of the diver from the LF-band and the LF/LH ratio, these did not show a significant increase at the beginning of the dives. This finding is in line with an earlier finding with experienced divers (Schipke and Pelzer, 2001). On the other hand, SNS activity is only one of the factors that contribute to the LF band. The significant increase in mean heart rate at the beginning of the dives suggests that there actually was a strong activation of the SNS. This is in line with most earlier observations of the diving reflex and sensation of cold also causing an SNS activation (Boussuges et al., 2007; Buchholz et al., 2017). After the first SNS response, the mean heart rate and LF/LH ratio suggested that SNS activity actually decreased over time. Our finding indicated that, for our experienced subjects, cold was, after the first responses to diving, neither a physiological nor a psychological stress factor. On the other hand, the dives were not deep nor demanding. Physical stress at the beginning of a cold-water dive, together with the diving reflex and cold stress-induced SNS activation, leads to a quick concurrent increase in both PNS and SNS activity. This, in turn, is a known risk factor for arrhythmia and sudden death (Buchholz et al., 2017; Kane and Davis, 2018). For this reason, at the beginning of a cold-water dive, we recommend an adaptation phase before tasks requiring physical stress. Furthermore, we recommend that special emphasis be placed on evaluating cardiovascular risk factors and incipient signs of heart disease for persons who dive in Arctic conditions. In the fit-to-dive evaluations of Naval divers, we recommend strict cardiovascular criteria.

Our study had some limitations. First, it was a field study, with results gathered during regular diving equipment development tests and not in a more controlled environment, such as in a wet chamber. However, as diving was performed under the ice cover, the weather did not affect diving conditions, which were constant during all dives.

Secondly, the number of divers and dives was limited in our study. However, a small amount of measurements is not unusual in similar experimental field studies made in extreme conditions. Even with this small number of dives, results were statistically significant.

Thirdly, for a better HRV baseline and for evaluating the ANS changes caused by the diving reflex, we recommend measuring 5-min resting values before diving, 5 min-values after immersion, 5-min values directly after submersion, and the next 5-min measure 5–10 min after submersion for future studies. In our study, we have taken the resting HRV baseline from when the divers were in bed in the mornings and in the evenings. These are not necessarily exact HRV resting values because possible sleep may influence HRV (Penzel et al., 2016; Balasubramanian et al., 2017).

Our results received in a limited number of shallow dives in resting individuals cannot automatically be extrapolated to all types of SCUBA diving. During open-sea diving, in addition to water immersion and cold exposure, divers face supplementary stressors such as heart–lung interaction, induced by breathing a hyperbaric mixture through the regulator and hyperoxia. The increase in ambient pressure at depth leads to an increase in both the work of breathing and the oxygen partial pressure. All these stressors have been recognized to influence ANS and HRV.

## Conclusion

The first PNS response as a result of the human diving reflex decreased quickly. Our interpretation of this finding is that the trigeminocardiac part of the reflex declined quickly.

Both cold and hyperbaric pressure contributing to parasympathetic activity increased up to 80 min when diving in very cold water under constant hyperbaric pressure. Our study did not determine whether the increase in parasympathetic activity will reach a plateau at some point, which is why we feel that possible adverse effects of strong parasympathetic activity should be considered on long and cold dives.

Although our small study involved cold-adapted, experienced divers, we suggest an adaptation phase before physical activity at the beginning of dives in very cold water in order to reduce the risk of arrhythmia. Furthermore, it is prudent for special emphasis on cardiovascular risk factors to be placed on pre-dive evaluation of potential cold-water divers.

## Data Availability Statement

The datasets generated for this study are available on request to the corresponding author.

## Ethics Statement

The studies involving human participants were reviewed and approved by Ethical Committee of Tampere University Hospital Nr 2/2018. The patients/participants provided their written informed consent to participate in this study.

## Author Contributions

RL planned the study, took part in the data analysis, and was the main writer of the manuscript. AR-S planned the study, supervised RL during the data analysis, and was a writer of the manuscript. TW was present at the data-gathering phase, planned the study, and was a writer of the manuscript. TO planned the study, helped and worked with the HRV method, and was a writer of the manuscript. KP planned the study, supervised RL during the data analysis, and was a writer of the manuscript.

## Conflict of Interest

The authors declare that the research was conducted in the absence of any commercial or financial relationships that could be construed as a potential conflict of interest.
