# Supplementary material for: Diving in the Arctic: Cold Water Immersion’s Effects on Heart Rate Variability in Navy Divers
Source: Front Physiol. 2020 Jan 31;10:1600. doi: 10.3389/fphys.2019.01600 (PMC7005786; doi:10.3389/fphys.2019.01600)
Supplement: Supplementary file 1 [file Table_1.docx]

| Diver (number) | Age (years) | Height (metres) | Weight (kg) | BMI (kg/m^2^) | Body Fat Mass (kg) | Body Muscle Mass (kg) |
| --- | --- | --- | --- | --- | --- | --- |
| 1 | 43 | 1.78 | 79.2 | 25 | 13.7 | 37.2 |
| 2 | 25 | 1.8 | 80.3 | 24.8 | 4.9 | 43.3 |
| 3 | 40 | 1.72 | 86.4 | 29.2 | 14.5 | 41.7 |
| 4 | 49 | 1.81 | 86.8 | 26.5 | 13 | 42.4 |

Table S1: Demographics of the subjects
